# A Novel Method for Detection of Phosphorylation in Single Cells by Surface Enhanced Raman Scattering (SERS) using Composite Organic-Inorganic Nanoparticles (COINs)

**DOI:** 10.1371/journal.pone.0005206

**Published:** 2009-04-15

**Authors:** Catherine M. Shachaf, Sailaja V. Elchuri, Ai Leen Koh, Jing Zhu, Lienchi N. Nguyen, Dennis J. Mitchell, Jingwu Zhang, Kenneth B. Swartz, Lei Sun, Selena Chan, Robert Sinclair, Garry P. Nolan

**Affiliations:** 1 Department of Microbiology & Immunology, Stanford University, Stanford, California, United States of America; 2 The Baxter Laboratory in Genetic Pharmacology, Stanford University, Stanford, California, United States of America; 3 Materials Science and Engineering, Stanford University, Stanford, California, United States of America; 4 Biomedical/Life Sciences, Digital Health Group, Intel Corporation, Santa Clara, California, United States of America; University of Arkansas for Medical Sciences, United States of America

## Abstract

**Background:**

Detection of single cell epitopes has been a mainstay of immunophenotyping for over three decades, primarily using fluorescence techniques for quantitation. Fluorescence has broad overlapping spectra, limiting multiplexing abilities.

**Methodology/Principal Findings:**

To expand upon current detection systems, we developed a novel method for multi-color immuno-detection in single cells using “Composite Organic-Inorganic Nanoparticles” (COINs) Raman nanoparticles. COINs are Surface-Enhanced Raman Scattering (SERS) nanoparticles, with unique Raman spectra. To measure Raman spectra in single cells, we constructed an automated, compact, low noise and sensitive Raman microscopy device (Integrated Raman BioAnalyzer). Using this technology, we detected proteins expressed on the surface in single cells that distinguish T-cells among human blood cells. Finally, we measured intracellular phosphorylation of Stat1 (Y701) and Stat6 (Y641), with results comparable to flow cytometry.

**Conclusions/Significance:**

Thus, we have demonstrated the practicality of applying COIN nanoparticles for measuring intracellular phosphorylation, offering new possibilities to expand on the current fluorescent technology used for immunoassays in single cells.

## Introduction

To better understand the processes occurring in abnormal cells compared to normal cells, there is an urgent need to improve the technology for simultaneous detection of multiple events in a single cell. When coupled with surface marker definitions of cell type, intracellular staining for phosphoproteins can be a powerful tool for understanding the biochemistry of primary cell samples. However, one rapidly reaches limits on the numbers of simultaneous measurements that can be deployed with fluorophore based approaches. To date, antibodies have been most commonly labeled with fluorescent molecules. The use of up to 17 different fluorescent molecules has been implemented by FACS [1], but as is well understood the often overlapping spectra of fluorophores requires compensation and becomes more difficult to carry out with each additional parameter added. Therefore, there is a need to develop molecules that overcome the limitations of fluorescence in multi-parameter detection. Raman scattering may allow the detection and specific attribution of a signal among several simultaneously measured signals and thereby exceed the limit of fluorescence emission overlap adjustment. A first step for implementing a Raman Spectral Flow Cytometer has recently been used for the detection and discrimination of several SER-tags [2], [3] and the report detailed here is complementary to those efforts.

Spontaneous Raman scattering is typically very weak, and enhancement is required to improve the spatial resolution of the Raman scattering signal. Surface Enhanced Raman Scattering (SERS) has been successful in enhancing Raman signals using the elements silver, gold, or copper [4]–[11]. Particles composed of such elements are specifically useful as enhancers of Raman signals, since their surface plasmons (containing valence electrons) are easily excited by laser light, and generate an electric field that can be transferred to nearby Raman active molecules. This results in an amplification of the Raman signal by 10^3^–10^14^ fold [12]–[14]. By using a variety of Raman labels with distinct Raman spectral fingerprints, it is thus possible to generate a library of SERS molecules. With a carefully selected set of library members, it is possible to deconvolute the Raman spectra to determine the contribution of each individual signature in a combination of spectra. Thus the nanoparticles may be used as a tool for multiple signal detection.

Berlin and colleagues (Intel Corporation) created a clusters of highly active nanoparticles SERS nanoparticles with highly enhanced Raman scatters [9]. These nanoparticles were termed “Composite Organic-Inorganic Nanoparticles” (COINs). The composites are coalesced silver nanoparticles with entrapped organic Raman labels. The COINs are coated with BSA to be biocompatible [9]. COIN clusters enhance the Raman signal by 10^4–5^ fold compared to single silver particles coated with Raman dye. This additional enhancement improves detection of Raman signal from COINs used in antibody-conjugated immunoassays. This signal enhancement allows detection of protein and protein modifications in single cells comparable to fluorescence technology. COINs can be functionalized by cross-linking to biological specificity reagents such as antibodies for use in immuno-detection.

Here we report the utility of SERS-based COIN nanoparticles as nanotags for immuno-detection in single cells, measuring epitopes on the surface of cells as well as induced intracellular phospho-epitopes. We demonstrate the ability to deconvolute the Raman spectra of two simultaneous measurements of phosphorylation events in a single cell. The software is capable of deconvoluting eight spectra readily. The signals detected by Raman spectroscopy are comparable to those measured by conventional flow methods. This study is a demonstration of the sensitivity of SERS-based COIN agents and their utility for detecting proteins and protein modifications, providing a roadmap for more flexibly to add new parameters in simultaneous measurement of biological events in single cells.

## Results

### Composite Organic-Inorganic Nanoparticles –COINs

Composite Organic-Inorganic Nanoparticles (COINs) with Surface Enhanced Raman Scattering (SERS) properties were fabricated as previously described [9]. The COIN clusters are silver nanoparticle aggregates initiated with either heat or salts in the presence of organic Raman dyes. In the context of this report, we elaborate on the fabrication and uses of two different COINs, fabricated using either of the methods with the Raman dyes: Acridine Orange, and Basic Fuchsin.

**Figure 1:**
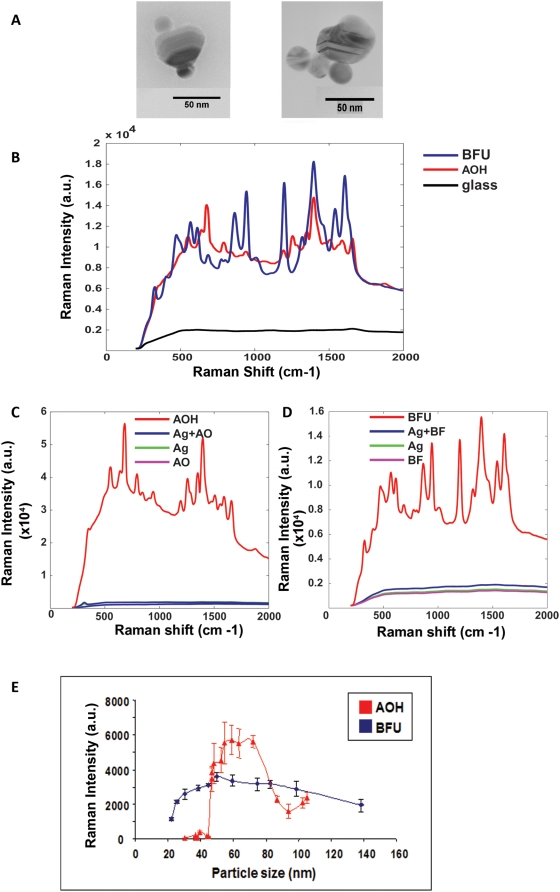
Characteristics of Composite Organic-Inorganic Nanoparticles COINS. a) Transmission electron microscopy images of COINs, AOH (left) and BFU (right). b) Spectral signature of AOH COIN (red), BFU COIN (blue) and glass background (black) are indicated as Raman intensity. c) COIN aggregates enhance the Raman signal of SERS particles. The Raman intensity was measured for the dye alone (AO), silver (Ag) silver+dye (Ag+AO) and COIN−AOH and d) BFU [dye alone (BF), silver (Ag), silver+dye (Ag+BF), and (COIN−BFU) respectively. e) Raman intensity signal of COIN compared to the size of COIN cluster measured for AOH (red) and BFU (blue).

The AOH COIN was fabricated by heating a 12 nm silver seed in the presence of Acridine Orange reduced with sodium citrate. This process generates random clusters of silver particles with a specific Raman signature **(** **Figure 1A,B** **)**. The BFU COIN was generated by first enlarging the silver seed to 24 nm and inducing aggregation in the presence of sodium chloride (NaCl) and Basic Fuchsin. The BFU COIN clusters are similar to the AOH COIN but have a different Raman signature **(** **Figure 1A,B** **)**. The Raman intensity of spectra for COINs was significantly enhanced by the generation of clusters. Mixing of the silver seed particles with the Raman dye generated colloid silver particles with non-detectable Raman shifts. However, the aggregation of the silver particles into COIN clusters, significantly enhanced the Raman signal intensity by approximately 10^4^–10^5^ fold. **(** **Figure 1C,D** **)**. To determine Raman activity related to COIN cluster size we generated COINs of increasing sizes. The nanoparticle size and polydispersity was determined using photon correlation spectroscopy (PCS: Zetasizer, Malvern). The crude COINs were scanned for their Raman spectra using IRBA (see following paragraph). We found that the intensity of the Raman spectra increases with the size of the COIN particles **(** **Figure 1E** **)**. The trend is different for the different COINs. The Raman intensity for the AOH COINs increased abruptly when the mean size grew beyond 50 nm, and the intensity decreased when the particle size grew beyond 80 nm. The increase of the Raman signal for the BFU COIN was moderate but reached optimal intensity between 50–60 nm and decreased beyond that size. The COIN size suitable for bioassays was determined to be 60±6 nm for AOH and 52±5 nm for BFU, where the optimal intensity of the Raman peak was observed for each COIN. Thus, we have generated SERS based COIN nanoparticles that have specific and enhanced Raman shifts.

### Raman microscopy

**Figure 2:**
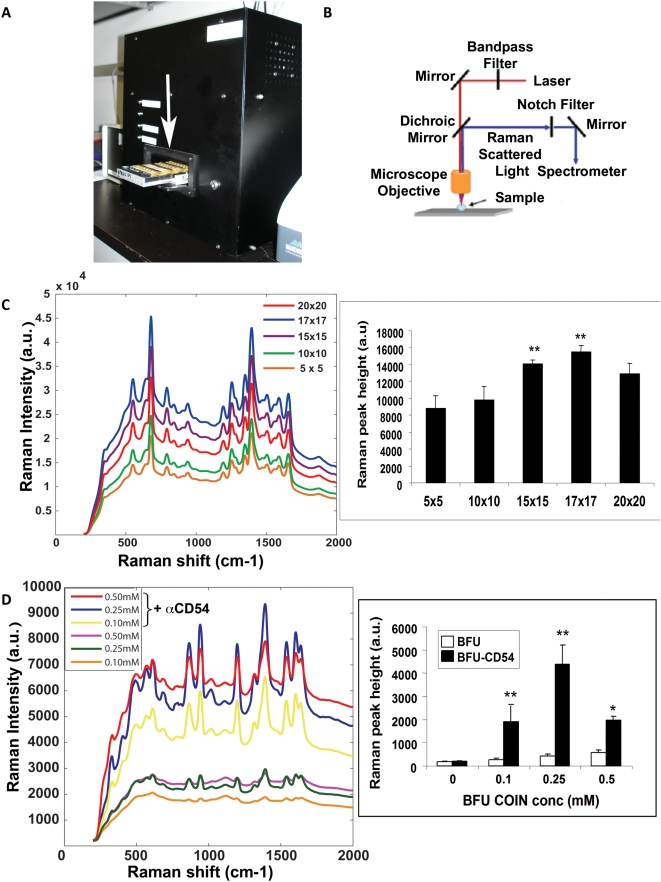
Raman microscopy. a) Image of the “Integrated Raman BioAnalyzer” - IRBA. The arrow indicates the placement of the chamber with the sample prior to insertion into the apparatus. b) Generic configuration of Raman microscopic setup. c) Optimization of scans using IRBA. Wells containing COINs were scanned with a laser beam of 1 µm using a matrices of 5×5, 10×10, 15×15, 17×17 and 20×20 at 100 µm distances. The spectra are indicated (left) and the calculated peak heights are represented as histograms (right). The experiments were performed 3 times in duplicates. The peak heights for the 15×15 and 17×17 are significantly different from the 5×5, 10×10 and 20×20; **p<0.01. d) Raman intensity of spectra from cells stained with different concentrations of αCD54-AOH-COIN (red - 0.5 mM, blue – 0.25 mM and yellow – 0.1 mM) and AOH-COIN (purple - 0.5 mM, green – 0.25 mM and orange – 0.1 mM), scanned by IRBA (left). Quantitation of the Raman peak height from the spectra observed illustrated as histograms *p<0.05 and **<0.01. The experiment was performed three times in duplicates.

To reliably detect the Raman signal in a format appropriate for cellular analyses, we developed a automated Raman scanner (Intel Raman BioAnalyser – IRBA) that is suitable for detecting Raman signals **(** **Figure 2A** **)**. The schema for the IRBA is illustrated in **(** **Figure 2B** **)**. The key components of the microscope are the dichroic filter and notch filter. The dichroic filter allows the laser light to reach the sample, and reflect all other wavelengths. The notch filter blocks the laser light, and transmits all other light wavelengths. The Raman scattering is measured as spectral shifts as little as 30 nm from the excitation laser-light source, hence the slope of the notch filter is high (∼90 degrees).

The IRBA scans 64-wells in a microtiter plate-like format. Biological specimens are immobilized on aldehyde glass slides, assembled into a FAST Frame slide holder adopting the 64-well footprint. The sample wells were filled with phosphate buffered saline (PBS), covered with cover glass and loaded into the sample tray holder of the IRBA (see arrow in Figure 2a). During the scan, samples were probed by a continuous wave, diode-pumped, solid-state laser. IRBA custom software is prompted to automatically focus the laser beam onto the sample using an aspheric objective lens with *f*/0.5 numerical aperture and a 20× magnification. The laser power at the sample stage is 100 mW, with a laser spot size ∼1 µm in diameter. A mechanical shutter reduces the sample exposure to laser light. A typical exposure time is 0.1 seconds per spot. The detector is a back-illuminated, thermoelectrically-cooled CCD camera. The IRBA custom software conducts automated data acquisition of the slide using a user-defined raster scan. The IRBA configuration is set up to collect a single Raman spectrum from a 1 micron spot at a distance of 10 microns with an acquisition time of 100 ms. The IRBA performs a raster scan of the sample containing wells, using a scan matrix of 2×2 up to 20×20 with 100 µm intervals. We tested the optimal raster scan using an AOH COIN solution. We found an increase in the Raman intensity signal with the increase in scan parameters. The optimal results were obtained using a scan matrix of 17×17 matrices with 100 µm intervals **(** **Figure 2C** **)**. Thus, the Raman scanner is able to scan a sample plated in a well-chamber. This is applicable to further analysis of cells, as detailed below.

### Detection of cell surface antigens using Raman COINs

We tested COINs in immunoassays. We first determined the ability to use COIN nanoparticles to detect surface antigens on single cells stained in suspension. Antibodies were conjugated to COINs. The ability for an antibody-conjugated COIN to function in a bioassay was first determined in an IL-8 ELISA sandwich assay **(Figure S1A)**. The IL-8 antibody-COIN conjugate that shows a linear reactivity to IL-8 antigen concentration with a linear slope (r^2^>0.8) is considered suitable for use in additional bioassays. Both the AOH and BFU COINs, representing two different fabrication processes, passed the initial control and were considered suitable for use in other biological assays.

**Figure 3:**
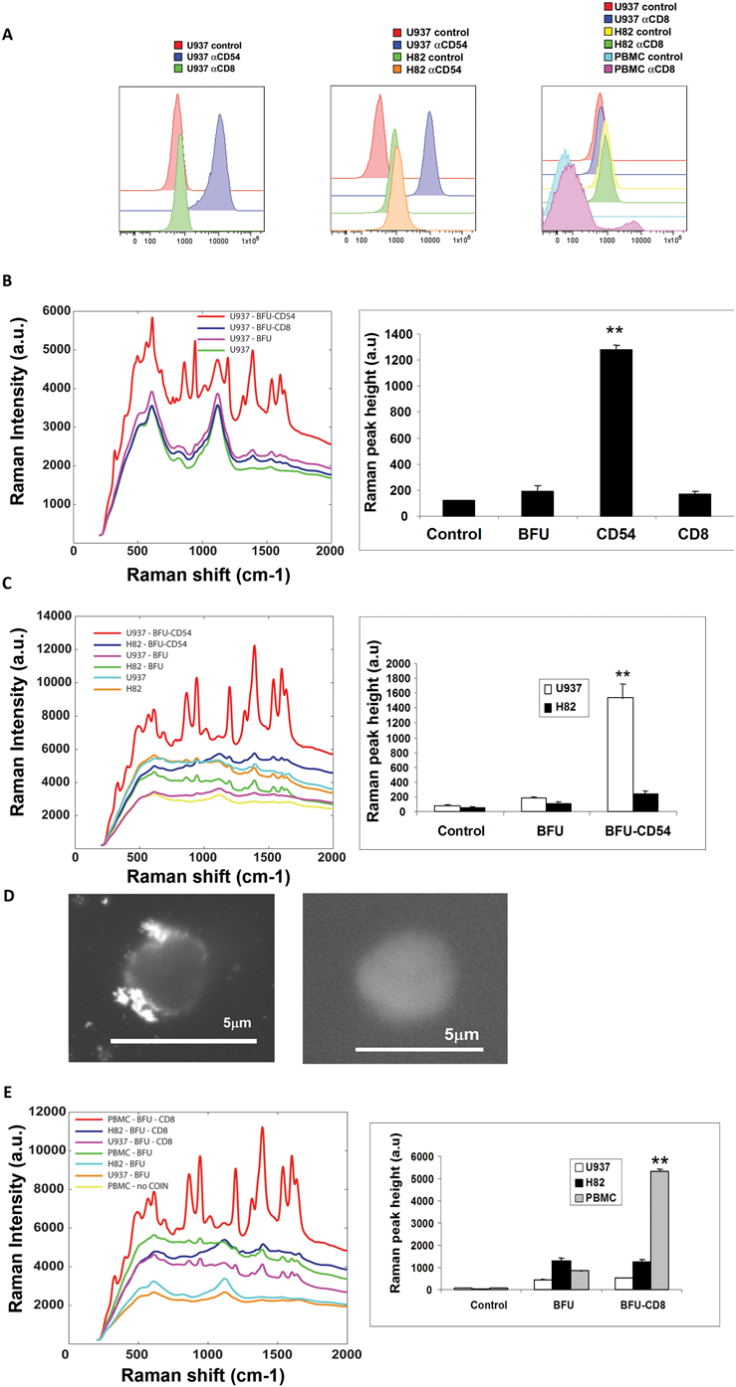
Specificity of COIN based Raman spectroscopy for detection of Surface antigens. a) Expression of ICAM (CD54) and absence of CD8 expression on U937 cells was determined by FLOW cytometry (left). Expression of CD54 on U937-expressing cells and H82-non-expressing cells was determined by FLOW cytometry (center). Expression of CD8 in a subset of human PBMCs was compared to non-expressing U937 and H82, determined by FLOW cytometry (right). b) Antigen specific detection of CD54 using COIN. Raman intensity of spectra from cells stained with αCD54-BFU and αCD8-BFU COINs (left). The spectra are representative for five independent experiments. Quantitation of Raman peak height is represented as histograms of five independent experiments performed in duplicates (right). The αCD54-BFU COINs specifically detected CD54 on U937 cells **p<0.01. c) Cell-specific detection of CD54 surface antigen using COIN. Raman spectra from CD54 expressing U937 cells and non-expressing H82 cells stained with αCD54-BFU COIN (left). Quantitation of Raman peak height is represented as histograms (right). The αCD54-BFU COINs specifically detected CD54 on U937 cells **p<0.01. d) SEM images of U937 cells stained with αCD54-BFU (left) and BFU (right) COINs. e) Characterization of a cell population in primary blood cells using COIN. Raman spectra of human PBMC, H82 and U937 cells stained with αCD8-BFU COIN (left). Quantitation of Raman peak height is represented as histograms (right). The αCD8-BFU COINs specifically detected CD8 on hPBMCs **p<0.01.

To further determine the utility of the COINs as detectors, we performed measurements of surface proteins expressed in the U937 cell line. The U937 cell line is a monocytic leukemia with high ICAM-1 (CD-54 adhesion molecule) expression on the cell surface (**Figure 3A** **,** left & center). The AOH and BFU COINs were conjugated with CD54 antibodies and used to detect the CD54 antigen in an ELISA **(Figure S1B)**. Linear regression analysis of COIN signal versus antigen concentration in the ELISA yielded correlation coefficients (r^2^) of 0.8–0.99. Thus both the AOH and BFU COINs were found suitable to be used in cell staining to analyze the antigen on the cell surface.

We determined the optimal concentration of the COIN in the surface staining protocol. Increasing concentrations (0.1, 0.25, 0.5 mM) of COIN+αCD54 were incubated with U-937 cells. Excess unbound COIN was washed off and 0.5×10^6^ cells were spun down in the scanning chamber wells. The chamber-containing cells were scanned using IRBA and the 17×17 scan protocol, previously determined as optimal. The average spectrum was calculated for the spectra acquired for each well (**Figure 2D**). Raman peaks for the COIN signal were defined, and peak heights were calculated **(Figure S2)**. The peak heights are displayed as histograms (**Figure 2D**). We found an increase in BFU-COIN specific peak height with an increase in concentration from 0.1 to 0.25 mM, and a decrease in peak height when COIN concentration increased to 0.5 mM. A similar trend was observed for the AOH COIN **(Figure S3)**. We determined that the optimal concentration for COIN staining for further experiments is 0.25 mM.

To determine the accuracy of COINs for detecting specific surface antigens, we first tested the ability of the COINs to bind to CD54 antigen expressed on U937 cells compared to CD8 antigen that is not expressed on U937 cells (**Figure 3A,** left panel). We acquired the spectra for cells stained with antibody conjugated COIN and non-conjugated COIN (**Figure 3B** **,** left). The peak heights for each spectrum were quantitated and represented as histograms (**Figure 3B** **,** right). The Raman peak ratios were determined for the relative Raman peak heights of antibody-conjugated COIN compared to non-conjugated COIN. We detected a specific reactivity of the αCD54-antibody conjugated COIN in U937 cells. Both the AOH and BFU COINs showed similar detection reactivity to CD54 on the surface of U937 cells **(Figure S4A)**. To determine the cell-specific binding of the COIN we also stained H82 small cell lung cancer (SCLC) cells that do not express CD54 (**Figure 3A** **,** middle panel). We found specific binding of the αCD54-COIN to CD54 expressing U937 cells but not to H82 cells (**Figure 3C**). The results using the BFU COIN are comparable to the AOH COIN **(Figure S4B)**.

To visualize the localization of CD54-COIN on the cell surface, we analyzed U937 cells by Scanning Electron Microscopy (SEM). We imaged the cells stained with COIN without additional processing, which is usually required for SEM, by using Quantomix capsules. Using SEM on native samples, we detected clusters of COINs at the apex of U937 cells, which is characteristic for the expression of CD54 (**Figure 3D**).

To determine the utility of COINs for staining primary human cells, we stained human peripheral blood mononuclear cells (PBMC) with αCD8-conjugated COINs. A subset (∼7%) of the total hPBMCs is CD8+ T-cells, as measured by flow cytometry (**Figure 3A** **,** right). We detected a αCD8-COIN signal in PBMC but not in either U937 or H82 cells **(** **Figure 3E** **)**. To determine if only a subset of the cells reacted to the αCD8-conjugated COIN, we examined each scan for Raman spectra. Approximately 10% of the scans yield Raman spectra correlating with specific COIN signals. This percentage of positive signals compares to the range of cells positive by FLOW cytometry. We repeated the stain, now using AOH COIN. The results received with the BFU COIN were comparable to the AOH COIN **(Figure S4C)**.

We conclude that antibody-conjugated COINs bind specifically to antigens when used for immunostaining of single cells. The intensity of the Raman peak height may vary for each COIN. However, the calculated Raman peak height ratio of the antibody-conjugated COIN compared to non-conjugated COIN was similar for both AOH and BFU. Thus, we have demonstrated the utility of COIN for staining cell lines as well as primary human samples.

### Detection of intracellular phosphorylation signaling using Raman COINs

**Figure 4:**
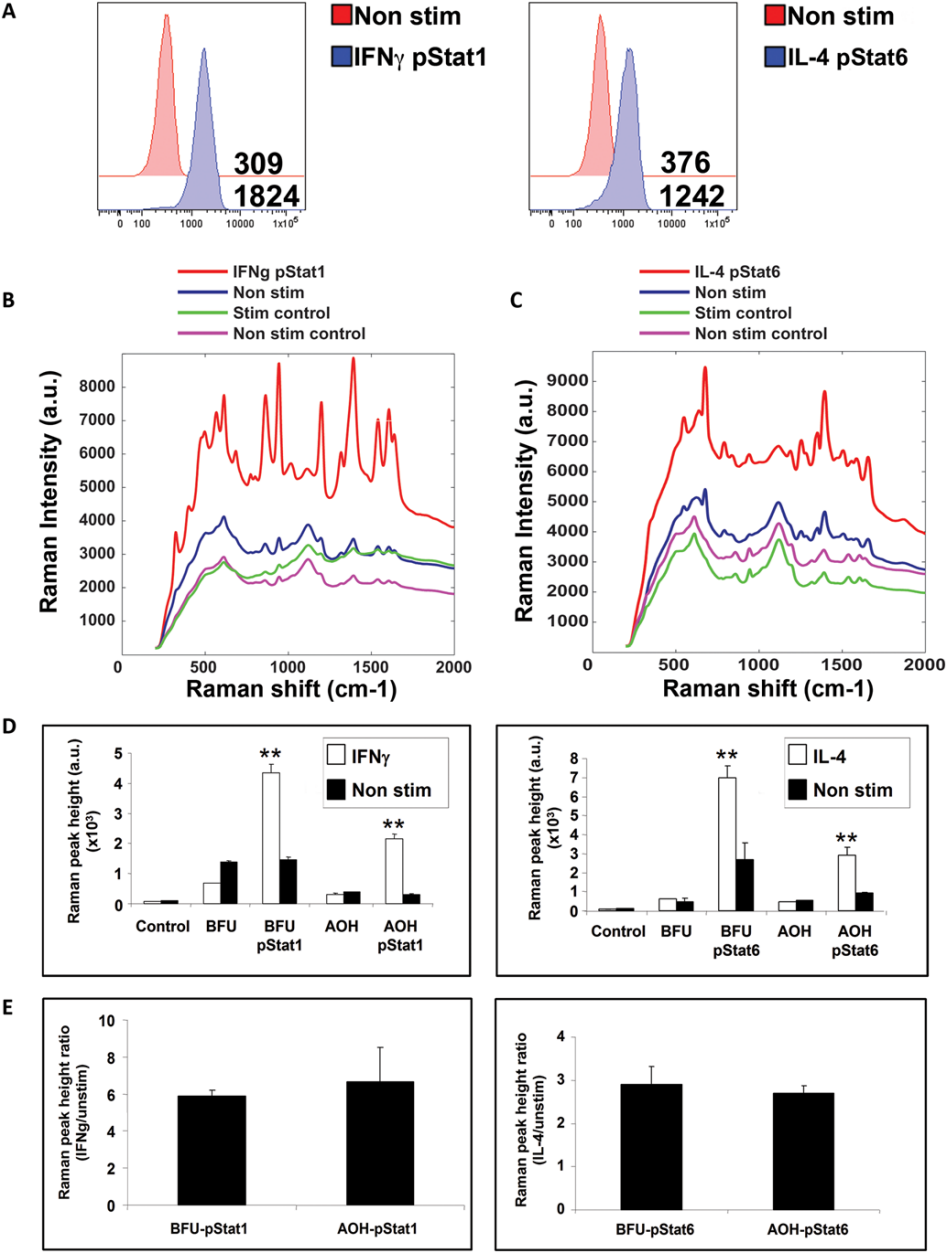
Detection of intracellular phosphorylation signaling using COINs. a) Flow analysis of pStat1 and Stat6 phosphorylation following treatment of U937 cells with IFNγ or IL-4 cytokines, compared to non treated (Non stim). b) Raman spectral shift intensity of BFU COIN detecting intracellular pStat1 in IFNγ and c) pStat6 in IL-4, treated and non-treated (Non stim) U937cells. Treated (Stim control) and non treated cells (Non stim control) were stained with non-conjugated BFU COIN. The spectra are representative for five independent experiments. d) Quantitation of change in Raman peak height after IFNγ and e) IL-4 treated cells compared to non-treated cells. The αpStat1 and αStat6 conjugated COINs specifically detected pStat1 and pStat6 respectively on IFNγ and IL-4 treated U937 cells compared to non-treated cells (**p<0.01). e) Fold change ratio of pStat1 and pStat6 phosphorylation in U937 treated cells stained with both BFU and AOH COINs. The changes are the average of five independent experiments.

Next, we tested the potential of COIN nanoparticles for the detection of intracellular phosphorylation events. U937 cells activate intracellular signal transduction pathways when treated with IL-4 and IFNγ. Treatment with IL-4 induces the phosphorylation of Stat6, while treatment with IFNγ induces the phosphorylation of Stat1. We first confirmed the increase in phosphorylation of Stat1 and Stat6 by PhosphoFlow analysis (**Figure 4A**). We measured a 5.9 fold increase of the phosphorylation of pStat1 following IFNγ treatment and 3.3 fold increase in phosphorylation of pStat6 following IL-4 treatment. BFU and AOH COINs were conjugated to antibodies that recognize the Y701 phosphorylated epitope of the Stat1, and the Y641 epitope of the Stat6 proteins. The cells were then fixed and permeabilized as previously described [15].

To prevent non-specific binding of COIN to intracellular proteins, an additional fixation step was carried out. Non-treated and treated cells were stained with antibody-conjugated and non-conjugated COIN washed and scanned using IRBA. The average spectra for IFNγ and IL-4 treated and non-treated cells are shown for AOH-pStat6 (**Figure 4D**) and BFU-pStat1 **(** **Figure 4C** **)**. To determine if the COIN itself affects the binding ability, the antibodies were alternated on each COIN. The changes in peak height were determined and the ratio of the Raman signal in treated cells was compared to non-treated cells **(** **Figure 4C** **)**. We detected a 5.9 fold change in pStat1 phosphorylation using αpStat1-BFU COIN and a 6.7 fold change using αpStat1-AOH COIN. We detected a 2.9 fold change in pStat6 phosphorylation using αpStat6-BFU COIN and a 2.7 fold change in using αpStat6-AOH COIN. The detected changes in phosphorylation of the Stat1 and Stat6 molecules using the AOH or the BFU COINs was similar to what was observed by PhosphoFlow.

Thus we have demonstrated the utility of COINs for measuring intracellular phosphorylation events in single cells.

### Detection of two simultaneous Raman signals using COINs

**Figure 5:**
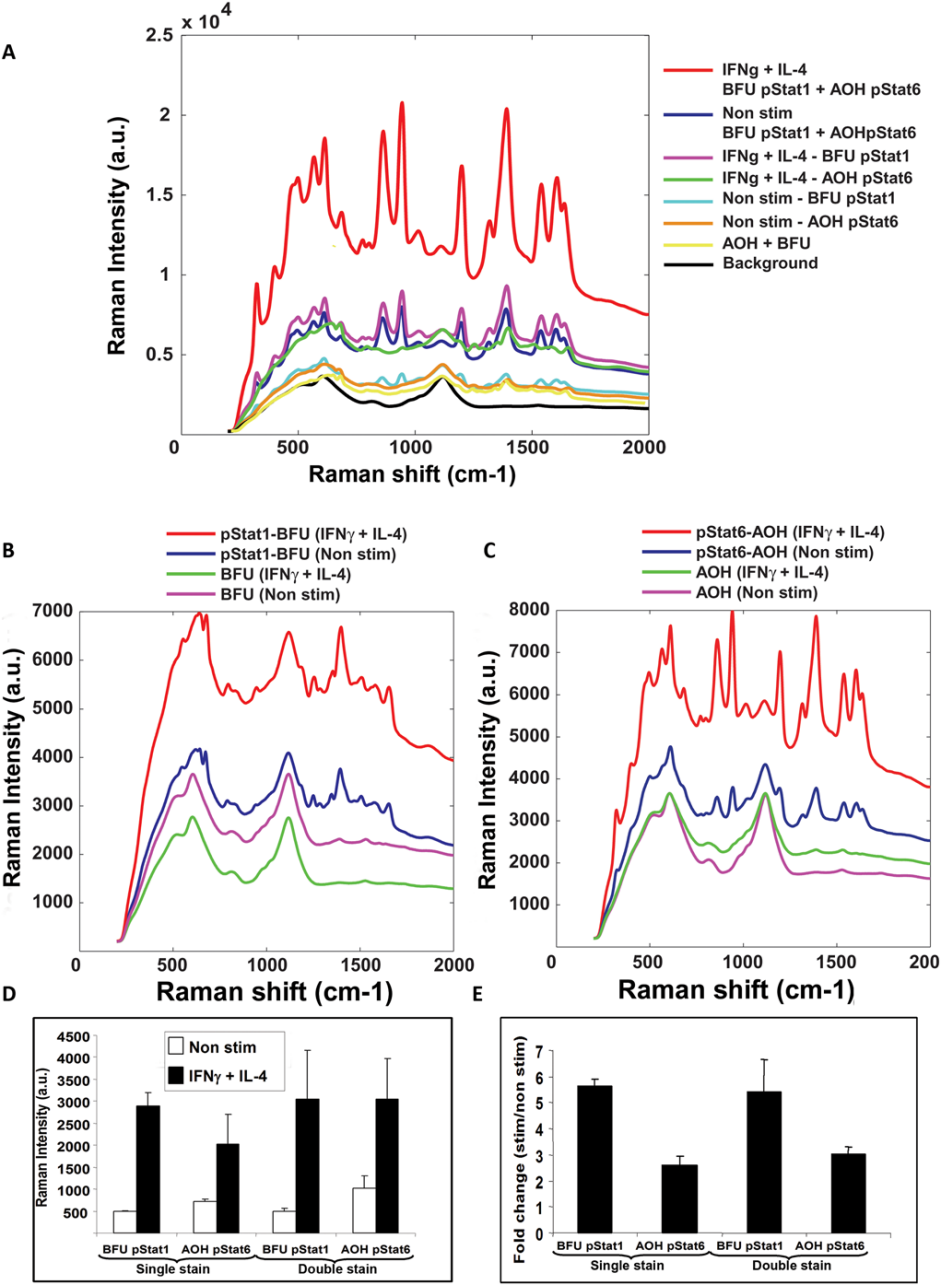
Detection of two intracellular phosphorylation events using two different COINs simultaneously. a) Raman spectra of the U937 cells treated with IFNγ and IL-4 simultaneously. The cells were stained with αpStat1-BFU and αpStat6-AOH simultaneously and separately. Cells were also stained with non-conjugated AOH and BFU COINs. The spectra are representative for five independent experiments. Cells were also stained with BFU and AOH that were not conjugated to antibodies. b) Extrapolated COIN spectra for treated (IFNγ+IL-4) and untreated cells (Non stim) stained with pStat1-BFU and BFU. c) Extrapolated COIN spectra for treated and untreated cells stained with pStat6-AOH and AOH. d) The Raman intensity of the Raman spectra for pStat1-BFU and pStat6-AOH COINs were calculated using the “MultiPlex” program (© Intel Corporation). The results are presented as histograms for single and double stain procedures. e) The fold change is the identified intensity of the spectra of the αpStat1 and αpStat6 conjugated COINs from treated and non-treated cells normalized to non-conjugated BFU and AOH COINs that were not conjugated to antibody. The results are the average of five independent experiments.

Ultimately, we determined the ability to conduct intracellular multiplex assays using COINs. A multi-parameter analysis was designed and simultaneous stained cells with AOH and BFU COINs, for detecting two phosphorylation events in a single cell. We co-treated U937 cells with IFNγ and IL-4. We conducted a simultaneous staining of the cells using BFU conjugated to pStat1 and AOH conjugated to pStat6 antibody. We also stained cells with BFU-pStat1, AOH-pStat6 and non-conjugated BFU and AOH COINs as controls. The cells were then scanned by IRBA and the Raman signal intensities detected from the samples are displayed **(** **Figure 5A** **)**. We used the “MultiPlex” program (© Intel Corporation) to deconvolute the two Raman spectra detected simultaneously from the BFU and AOH COINs. The representative Raman spectra for each COIN were identified and deconvoluted. We then extracted spectra for untreated cells, treated cells for the pStat1-BFU and pStat6-AOH COINs **(** **Figure 5B,C** **)**. Peak heights representative for each spectrum, were measured and the changes in ratio of the antibody-conjugated COIN peaks were compared to non-conjugated COIN, in treated and non-treated cells **(** **Figure 5D** **)**. The results from the double assay were also compared to the single assay in the experimental setup. We measured a 5.4 fold increase in pStat1 in the double stain compared to 5.7 fold change in the single stain experiment. We measured a 3.1 fold increase in pStat6 in the double stain compared to a 2.9 fold change in the single stain experiment. The calculated changes in peak height ratio were statistically similar when we used two COINs simultaneously compared to using a single COIN in a staining assay.

To illustrate the robustness of simultaneous staining procedures for phospho-epitopes with COINs, we treated cells with IFNγ (pStat1) or IL-4 (pStat6) or IFNγ/IL-4 (pStat1/pStat6). We stained the cell samples simultaneously with both COINs; the BFU COIN conjugated to pStat1 and the AOH COIN conjugated to pStat6 antibody. The samples were scanned using IRBA. The Raman spectra were deconvoluted using a software package designed for the dataset, termed MultiPlex (©Intel Corporation). The Raman peak heights were calculated and represented as histograms **(Figure S5A)**. The peak heights from cells stained with antibody conjugated COINs were normalized to the Raman signal from cells stained with non-conjugated COINs **(Figure S5B)**. The Raman signal from cells treated with either IFNγ or IL-4 cytokine is statistically similar to the signal from cells stained with both cytokines simultaneously (p>0.2).

The data demonstrate it is possible to use COINs for the measurements of two simultaneous phosphorylation events in a cell staining assay.

## Discussion

In this study we have demonstrated the ability to use COIN nanoparticles for multi-plex immuno-detection in single cells. We can generate multiple and distinct COIN Raman nanoparticles with resolvable signatures that can be used to detect surface antigens and to measure changes in intracellular phosphorylation events. Enhanced Raman signatures via SERS and COIN technology, offers possibilities exceeding fluorescent dye technology limits. COIN Raman spectra have several sharp peaks that define a “fingerprint” for each COIN. We can readily deconvolute multiple COIN spectra and may easily exceed the limit of fluorescence technology in multi-detection assays (we have already deconvoluted up to eight spectra using simulated complex spectra, unpublished). The detection of Raman signal of COINs, whose Raman detection is independent of fluorescence, may provide a dramatic increase in the multiplicity of simultaneous measurements. Another advantage of Raman COIN technology is versatility. The Raman spectra of COINs are measured as a shift relative to the excitation wavelength. The excitation of fluorophores is confined to a specific wavelength and re-emits energy at different (but very specific) wavelengths. COIN can be excited by different wavelengths depending on the available equipment.

Recently developed cadmium selenide-based quantum dots (Q-dots) are used to complement fluorescent dyes. Q-dots are physically uniform, may be excited by a wide band of light and emit a narrow band of light depending on its size. Since the emission spectra of the quantum dots are narrower than fluorescent molecules, the spectral overlap is less than with fluorescence molecules and thus require less manipulation than fluorescence when used for immunoassays [1]. To date, eight different quantum dots are available. The ability to use Q-dots as fluorescent tags in combination with molecular dyes to tag antibodies has advanced our ability to measure several simultaneous events in a single cell setting the current high bar for flow cytometry at 17 simultaneous measurements per cell. The future goal of our work with COINs is to develop a technology for flow cytometry that can concurrently measure Raman spectra. We hope to integrate the Raman spectra detection with conventional flow cytometry to exceed the current limits of fluorescence technology.

For the purposes of this report, we developed an automatic scanner to acquire Raman spectra – the IRBA. The IRBA is a compact, bench-top instrument. It was formatted to scan samples in 64-well microtiter plate format with user-friendly custom software. The IRBA has a fully-enclosed laser system. The components were systematically integrated and optimized to meet the specifications of the intended experiments and target applications. The IRBA allowed the sampling of Raman spectra from single cells to generate data for further analysis and stands as a training platform for the development of future generations of machines, including those with true flow capabilities.

In conclusion, Raman COIN technology is emerging as a powerful tool that will be useful for multi-parameter simultaneous measurements of protein and protein modification occurring in a single cell. By enhancing our capacity to measure intracellular phospho specific events at the single cell level this will allow determinations of additional parameters in studies of cellular processes. Thus, studies that use intracellular potentiation as a marker of biochemical process [16]–[18], clinical outcome in primary patient materials [19]–[23], or for determinations of signaling networks by computational processes [24] can be enhanced.

## Materials and Methods

### Coin Fabrication

BFU and AOH COINs were fabricated at Intel and Stanford as previously described [9]. Briefly, for AOH COIN fabrication, 12 nm silver seeds were prepared with silver nitrate (AgNO_3_) and reduced by sodium borohydride (NaBH_4_). The silver seeds were then mixed with sodium citrate (Na_3_C_6_H_5_O_7)_ and 5–30 µM Acridine Orange Raman dye. The solution was heated at 95°C for 60 min during which seed particles grew with the adsorption of the Raman dye. The reaction was stopped by the addition of 0.5% Bovine Serum Albumin (BSA) (Roche, #10 238 040001).

BFU COIN was fabricated using Basic Fucshin as Raman dye. The silver seeds were heated at 95°C with 0.5 M AgNO_3_ and Na_3_C_6_H_5_O for 3 hrs. COIN clusters formed in the presence of 0.5 mM Basic Fucshin dye and 20 mM NaCl during a reaction time of 4 minutes. The process was stopped by the addition of 0.5% BSA. The COIN clusters are encapsulated with BSA to stabilize and to introduce bio-functional groups on the surface.

### COIN conjugation to antibodies

We conjugated the antibodies to the BSA encapsulation of the COINs as previously described [25]. The carboxylic groups on BSA are activated with N-(3-(dimethylamino)-propyl)-N/-ethylcarbodiimide (EDC) (Sigma, #39391). Antibodies used for COIN conjugation are: CD54 (BD, #550302), CD8 (BD Bioscience, 554716), pStat1 (Y701) (BD Biosciences, #612596), pStat6 (Y641) (BD Biosciences, #612600).

### IL8 antibody ELISA sandwich assay

We performed an ELISA immuno-quality control assay (iQC) using IL8 antibody to test the quality of COIN-antibody conjugate. We coated aldehyde treated slides (NUNC™, #23164) with IL8 capture antibody (BD Pharmingen, #554716), mounted on FAST® frames (Whatman Inc., #10486 001). We added 1–100 ng of IL8 to the wells for 15 minutes and then washed with PBST (×2). We used BFU or AOH COINs conjugated to αIL8 antibody (BD Pharmingen, #554717) to stain the wells for 1 hour at room temperature (RT). We washed the wells in PBST (PBS and 0.1% Tween 20) and 0.1 M NaCl. The wells were filled with PBS and covered with cover glass (VWR International, #48366 067). We measured the Raman spectra for each well, using the Integrated Raman BioAnalyzer (IRBA), and a 532 nm excitation laser. The COINs that pass the iQC criteria were used for further detection assays. The criteria are: 1) experimentally-derived linear relationship between IL-8 concentration and Raman intensity readings (r^2^ = 0.8–1); 2) the COIN should not precipitate during antibody conjugation.

### CD54 COIN ELISA direct binding assay

A CD54-COIN ELISA direct-binding assay was performed as described above using monoclonal αCD-54 antibody (BD Pharmingen, #555364) conjugated to AOH or BFU COINs. Wells were coated with 5 ng/ml- 500 ng/ml recombinant human CD-54 (I-CAM-1) protein (R&D, #ADP4-200). An experimentally-derived linear relationship between CD54 protein concentration and αCD54-COIN Raman intensity readings (r^2^ = 0.8–1) was used to determine that the COINs passed iQC. These antibody-COIN conjugates were used for further cell staining procedures.

### pStat-1 COIN ELISA sandwich assay

We performed the pStat1 COIN sandwich assay as described above. Rabbit monoclonal αStat-1 antibody (Cell Signaling Technologies, #9175) was used as the capture antibody. We incubated 0–10 µg pStat1 blocking peptide (Cell Signaling Technologies, #1038) in the antibody coated wells. We purified pStat1 (pY701) mouse monoclonal antibody (BD BioScience, #612233,) using Protein G and Protein A orientation kits (PIERCE, #44990), then conjugated the antibody to the AOH or BFU COINs. An experimentally-derived linear relationship between pStat1 peptide concentration and αpStat1-COIN Raman intensity readings (r^2^ = 0.8–1) was used to determine that the COINs passed iQC. These antibody-COIN conjugates were used for further cell staining procedures.

### Measurement of Raman signal using IRBA

We measured COIN Raman spectra using the IRBA and a 532 nm excitation laser. We used raster scan matrices: 5×5, 10×10, 15×15, 17×17 and 20×20 at 1 µm intervals to test Raman intensity signal acquisition. The 17×17 matrix scan (average of 289 scans) gave maximum Raman signal intensity and was therefore used for subsequent scanning. Raman spectra files acquired by IRBA were exported as text files. Peak height was determined using the PeakHeight software (© Intel Corporation) in MATLAB (The MathWorks, Inc). The peak height area was calculated using the following parameters: peak-start, peak-top and peak-end for each spectrum.

### Staining procedure for the detection of surface proteins by COINs

We cultured U937 cells (ATCC-CRL-1593.2) in RPMI medium (Invitrogen, Carlsbad, CA). We isolated hPBMCs using density gradient solution (Ficoll-Paque Plus; Amersham Biosciences). We washed cells in PBS and fixed in 1.5% paraformaldehyde (Electron Microscopy Sciences, Hatfield) for 15 minutes. We washed the cells in PBS then blocked them with 1% BSA (Fraction V (Sigma, #A4503) for 1 hour during rotation. We washed the cells in PBS (×1) and COIN staining buffer (×1) (PBST (PBS and 0.1% Tween 20)+10% fetal bovine serum (HyClone). We stained 2×10^6^ cells in 200 µl COIN staining buffer with 0.1, 0.25, and 0.5 mM concentration of COINs. The 0.25 mM concentration gave the best staining results and became the standard for our staining protocols. We washed the stained cells with PBST (×2) and then with PBS (×1) to remove the detergent. We immobilized 0.5×10^6^ cells by centrifugation at 1800g for 15 min on 0.5% gelatin coated aldehyde slides (G7765, Sigma) fixed on FAST® frames (Whatman Inc., #10486 001). We removed the supernatant, from the wells, replaced it with 200 µl PBS and covered the wells with cover glass (VWR International, #48366 067). We measured the Raman spectra using the IRBA and a 532 nm excitation laser.

### Detection of intracellular proteins by COINS

We treated U937 cells with human IFNγ (Peprotech, #200-04) and human IL-4 (Peprotech, #300-02) as previously described [15]. We suspended U937 cells in RPMI media at the concentration of 5×10^6^ cells/ml. We treated the cells for 15 minutes at 37°C with 20 ng/ml IFNγ to induce Stat1 phosphorylation or 20 ng/ml IL-4 to induce pStat6 phosphorylation. We fixed the cells in 1.5% PFA for 15 minutes, washed the cells in PBS, suspended them in 70% ethanol, and stored them at −80°C. Before staining with COIN, we washed the cells in PBS and fixed them in 1.5% PFA for 15 minutes at RT. We used the same staining protocol used for the detection of surface proteins described above.

### Raman signal unmixing

We treated the U937 cells in three ways: with human IFNγ, with human IL-4, or with both IFNγ and IL4 for the multiplex COIN analysis. We used the separately treated cells for different staining combinations. We stained some cells with both BFU-pStat1 and AOH-pStat6 COINs. We stained other cells with BFU-pStat1 or AOH-pStat6 only. We measured the Raman signal intensity of cells using the IRBA and a 532 nm excitation laser. We compared the Raman signals from cells stained with a single COIN to those of cells stained with both COINs. We repeated all staining combinations with non-treated cells as controls. We deconvoluted the spectra measured from samples stained with both COINS using the MultiPlex COIN analysis program (© Intel Corporation) run in MATLAB (The MathWorks, Inc). This analysis program uses the “Least Squares Method” to determine the spectral contribution from the different sources.

## Supporting Information

Figure S1 ELISA sandwich assay for the detection of surface antigens using COIN based Raman spectroscopy. a) ELISA sandwich assay of αIL-8-AOH (left) and αIL-8-BFU (right). b) ELISA assay of αCD54-AOH (left) and αCD54-BFU (right). c) Comparison of concentration dependence and staining performance of AOH and BFU COIN.
(1.53 MB TIF)

Figure S2 Peak height analysis. A representative peak is selected and the peak start, top and end are determined. The Raman peak is identified and projected to all spectra in the scans performed by IRBA. The area under the peak is determined for each sample and determined as peak height.
(1.00 MB DOC)

Figure S3 Optimization of COIN concentration in staining protocol. a) Quantitation of the Raman peak height from the spectra observed for cells stained with different αCD54-BFU-COIN and BFU-COIN concentrations, scanned using IRBA illustrated as histograms *p<0.05 and **<0.01. b) Comparison of concentration dependence of BFU and AOH COINs conjugated to αCD54. The fold change is the average of five independent experiments. There is no statistical difference between the BFU and AOH COINs (p>0.2).
(1.18 MB DOC)

Figure S4 Correlation between BFU and AOH COINs for surface antigen detection. a) Antigen specific detection of CD54 with COIN. Raman spectra quantitation of cells stained with αCD54-AOH and αCD8-AOH COINs represented as histograms (left) and is the average of five independent experiments. Specificity of αCD54-AOH in U937 cells is indicated (**p<0.01). Comparison of the Raman peak height ratio detected for the BFU and AOH COINs of CD54 and CD8 expression in U937 cells (right). b) Cell specific detection of CD54 surface antigen with AOH COIN. Raman spectra peak height quantitation of CD54 expressing U937 cells and non-expressing H82 cells stained with αCD54-AOH COIN is represented as histograms (left) and is the average of five independent experiments. Specificity of αCD54-AOH in U937 cells is indicated (**p<0.01). Comparison of the Raman peak height ratio detected for the BFU and AOH COINs of CD54 in U937 and H82 cells (right). c) Raman spectra peak height quantitation of human PBMC, H82 and U937 cells stained with αCD8-AOH COIN represented as histograms (left) and is the average of five independent experiments. Specificity of αCD8-AOH COINs is indicated (**p<0.01). Comparison of the Raman peak height ratio detected for the BFU and AOH COINs of CD8 expression in U937, H82 and human PBMC cells (right). The detection efficacy with BFU and AOH COINs are similar and not statistically different (p>0.2) (right).
(1.78 MB DOC)

Figure S5 (1.28 MB TIF)
