# Supplementary figures and images for: A Novel Method for Detection of Phosphorylation in Single Cells by Surface Enhanced Raman Scattering (SERS) using Composite Organic-Inorganic Nanoparticles (COINs)
Source: PLoS One. 2009 Apr 15;4(4):e5206. doi: 10.1371/journal.pone.0005206 (PMC2666268; doi:10.1371/journal.pone.0005206)

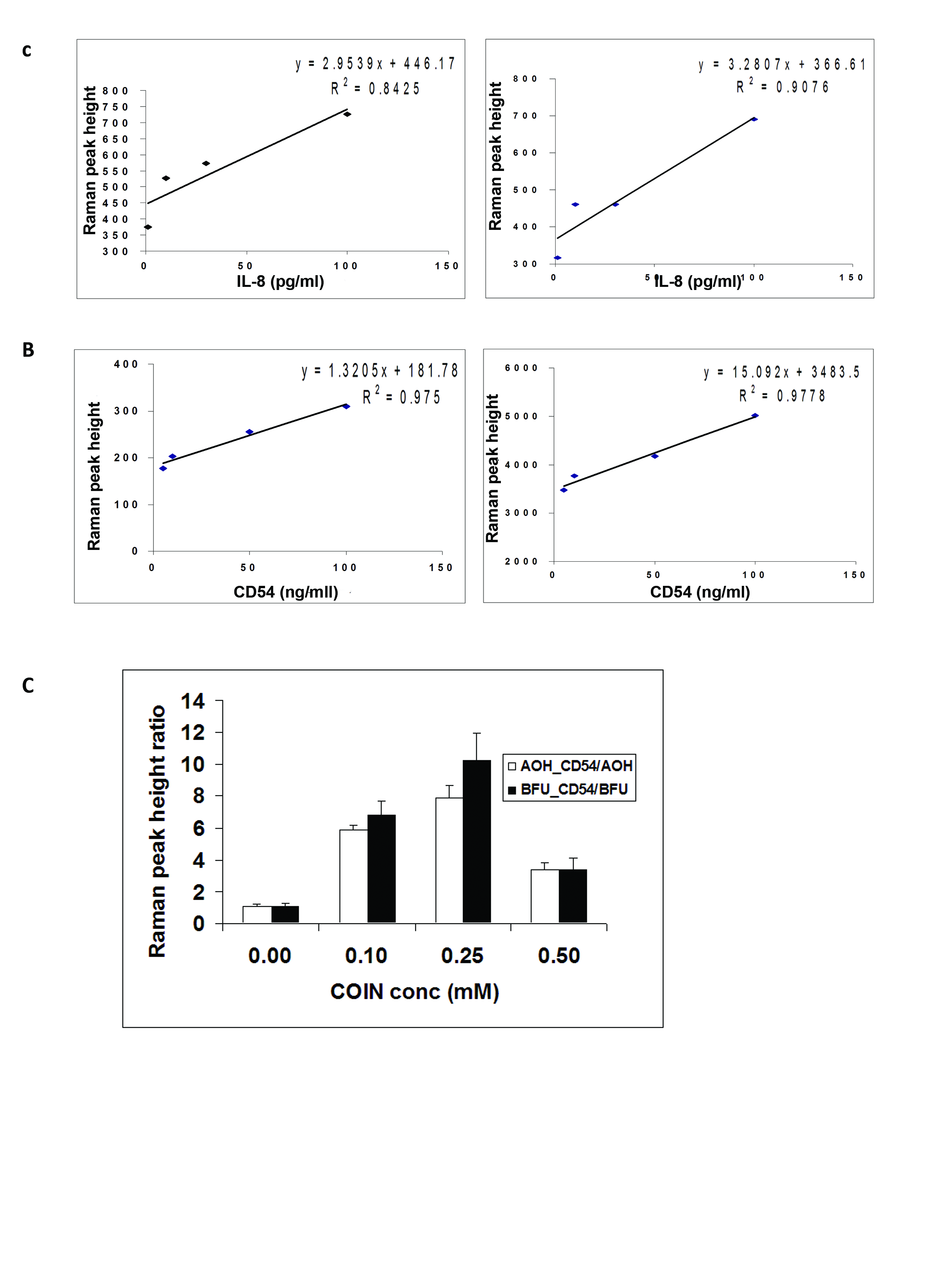

Supplement: Figure S1 — ELISA sandwich assay for the detection of surface antigens using COIN based Raman spectroscopy. a) ELISA sandwich assay of αIL-8-AOH (left) and αIL-8-BFU (right). b) ELISA assay of αCD54-AOH (left) and αCD54-BFU (right). c) Comparison of concentration dependence and staining performance of AOH and BFU COIN. (1.53 MB TIF) [file pone.0005206.s001.tif › Figure S1.tif]

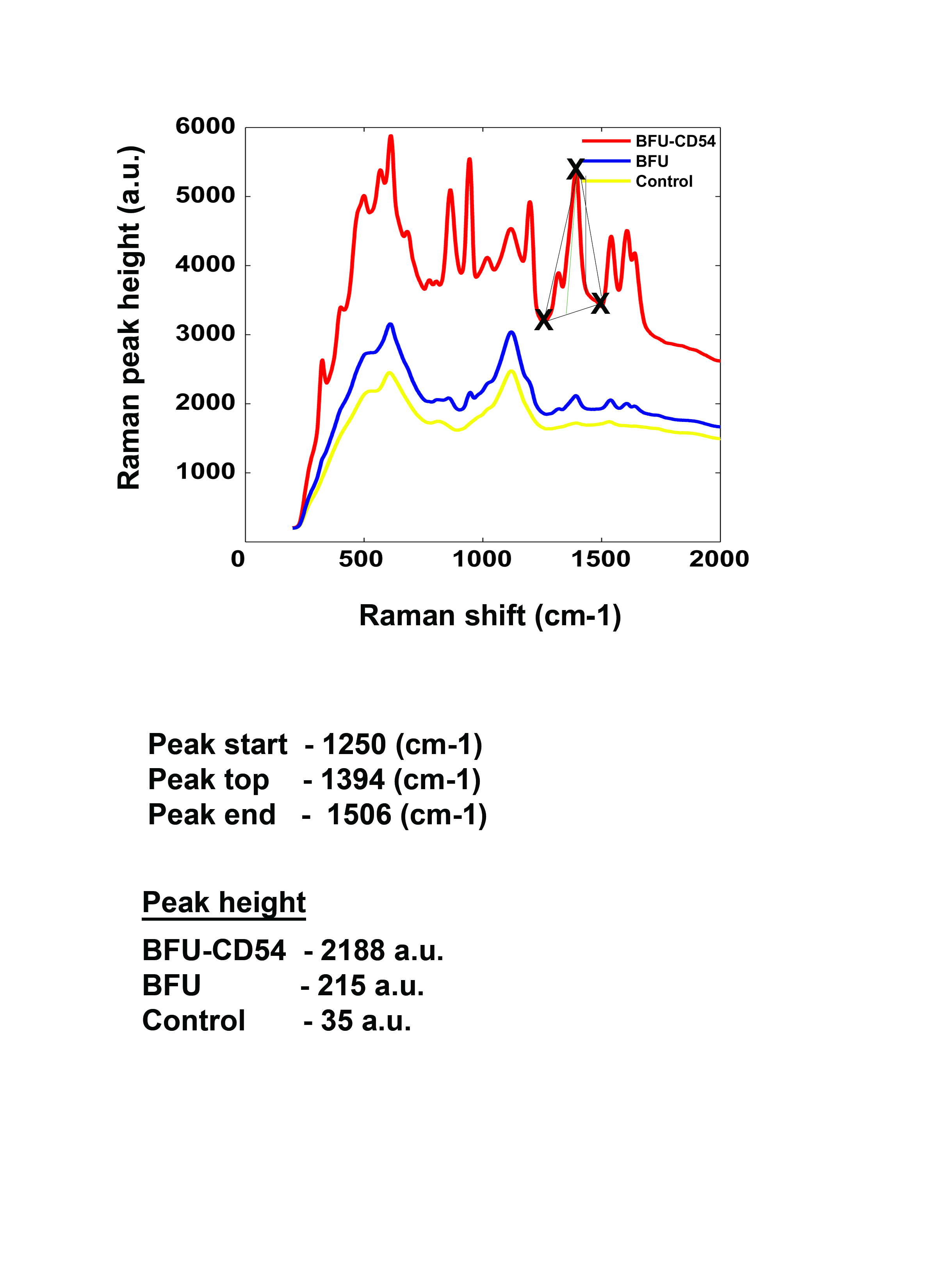

Supplement: Figure S2 — Peak height analysis. A representative peak is selected and the peak start, top and end are determined. The Raman peak is identified and projected to all spectra in the scans performed by IRBA. The area under the peak is determined for each sample and determined as peak height. (1.00 MB DOC) [file pone.0005206.s002.doc]

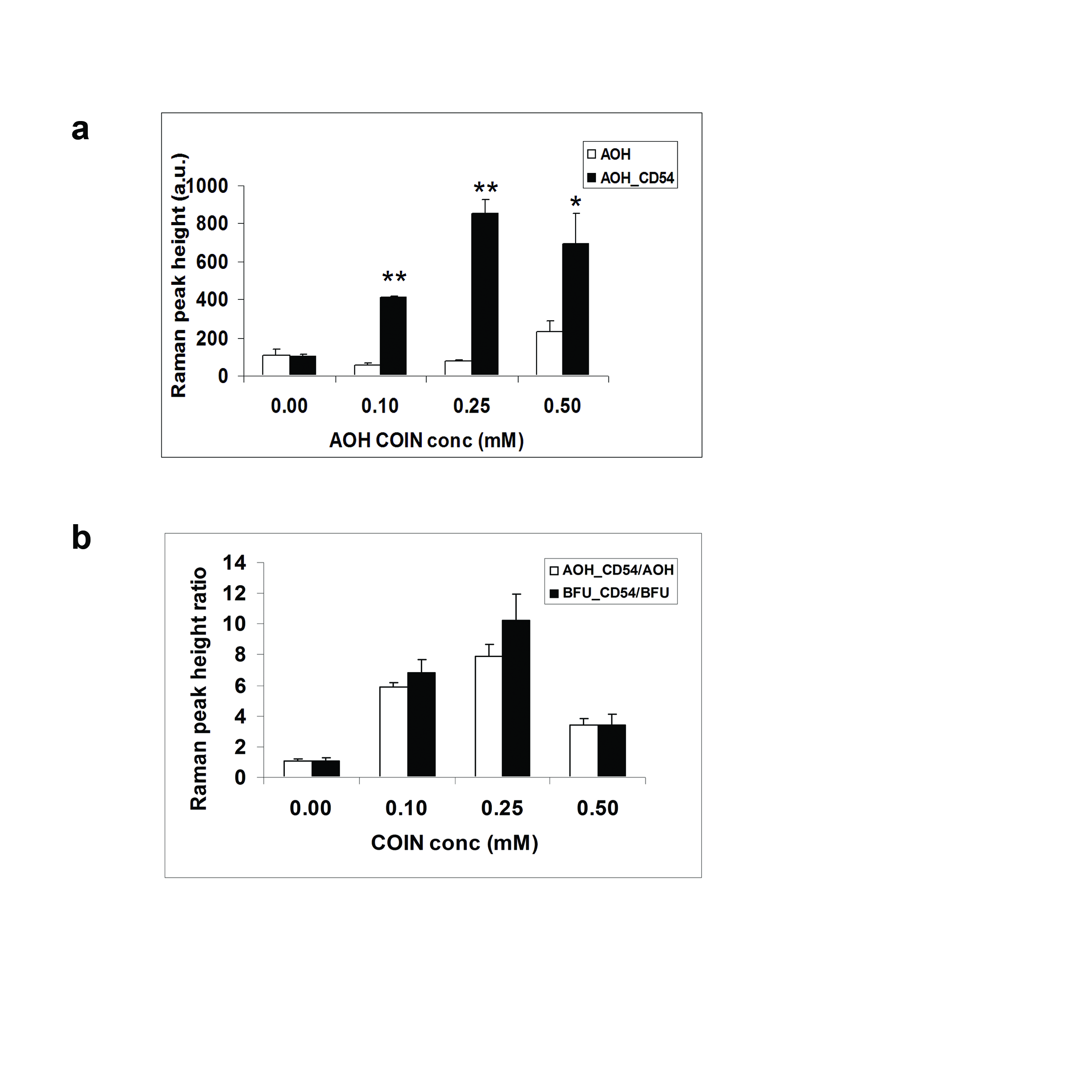

Supplement: Figure S3 — Optimization of COIN concentration in staining protocol. a) Quantitation of the Raman peak height from the spectra observed for cells stained with different αCD54-BFU-COIN and BFU-COIN concentrations, scanned using IRBA illustrated as histograms *p<0.05 and **<0.01. b) Comparison of concentration dependence of BFU and AOH COINs conjugated to αCD54. The fold change is the average of five independent experiments. There is no statistical difference between the BFU and AOH COINs (p>0.2). (1.18 MB DOC) [file pone.0005206.s003.doc]

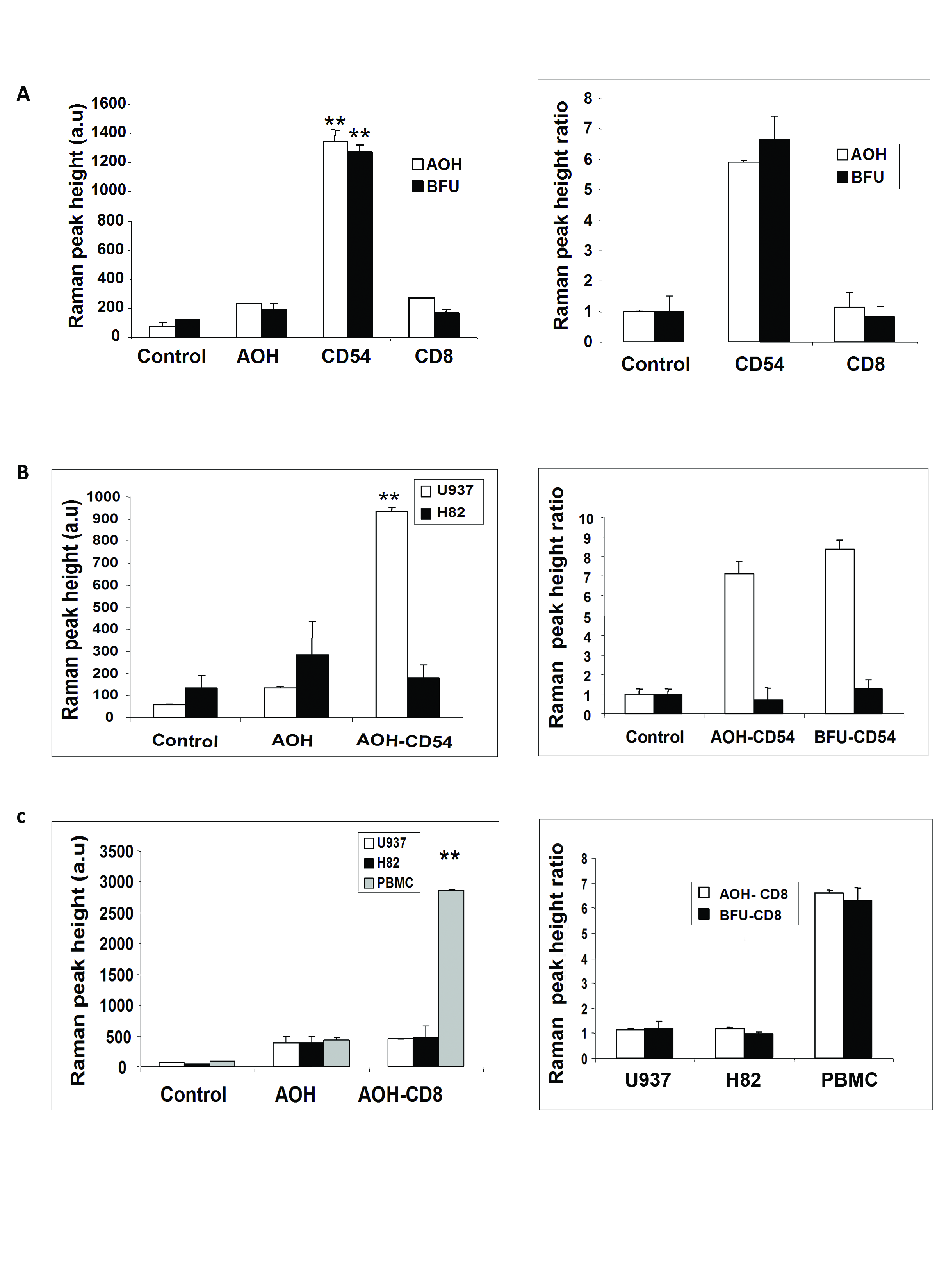

Supplement: Figure S4 — Correlation between BFU and AOH COINs for surface antigen detection. a) Antigen specific detection of CD54 with COIN. Raman spectra quantitation of cells stained with αCD54-AOH and αCD8-AOH COINs represented as histograms (left) and is the average of five independent experiments. Specificity of αCD54-AOH in U937 cells is indicated (**p<0.01). Comparison of the Raman peak height ratio detected for the BFU and AOH COINs of CD54 and CD8 expression in U937 cells (right). b) Cell specific detection of CD54 surface antigen with AOH COIN. Raman spectra peak height quantitation of CD54 expressing U937 cells and non-expressing H82 cells stained with αCD54-AOH COIN is represented as histograms (left) and is the average of five independent experiments. Specificity of αCD54-AOH in U937 cells is indicated (**p<0.01). Comparison of the Raman peak height ratio detected for the BFU and AOH COINs of CD54 in U937 and H82 cells (right). c) Raman spectra peak height quantitation of human PBMC, H82 and U937 cells stained with αCD8-AOH COIN represented as histograms (left) and is the average of five independent experiments. Specificity of αCD8-AOH COINs is indicated (**p<0.01). Comparison of the Raman peak height ratio detected for the BFU and AOH COINs of CD8 expression in U937, H82 and human PBMC cells (right). The detection efficacy with BFU and AOH COINs are similar and not statistically different (p>0.2) (right). (1.78 MB DOC) [file pone.0005206.s004.doc]

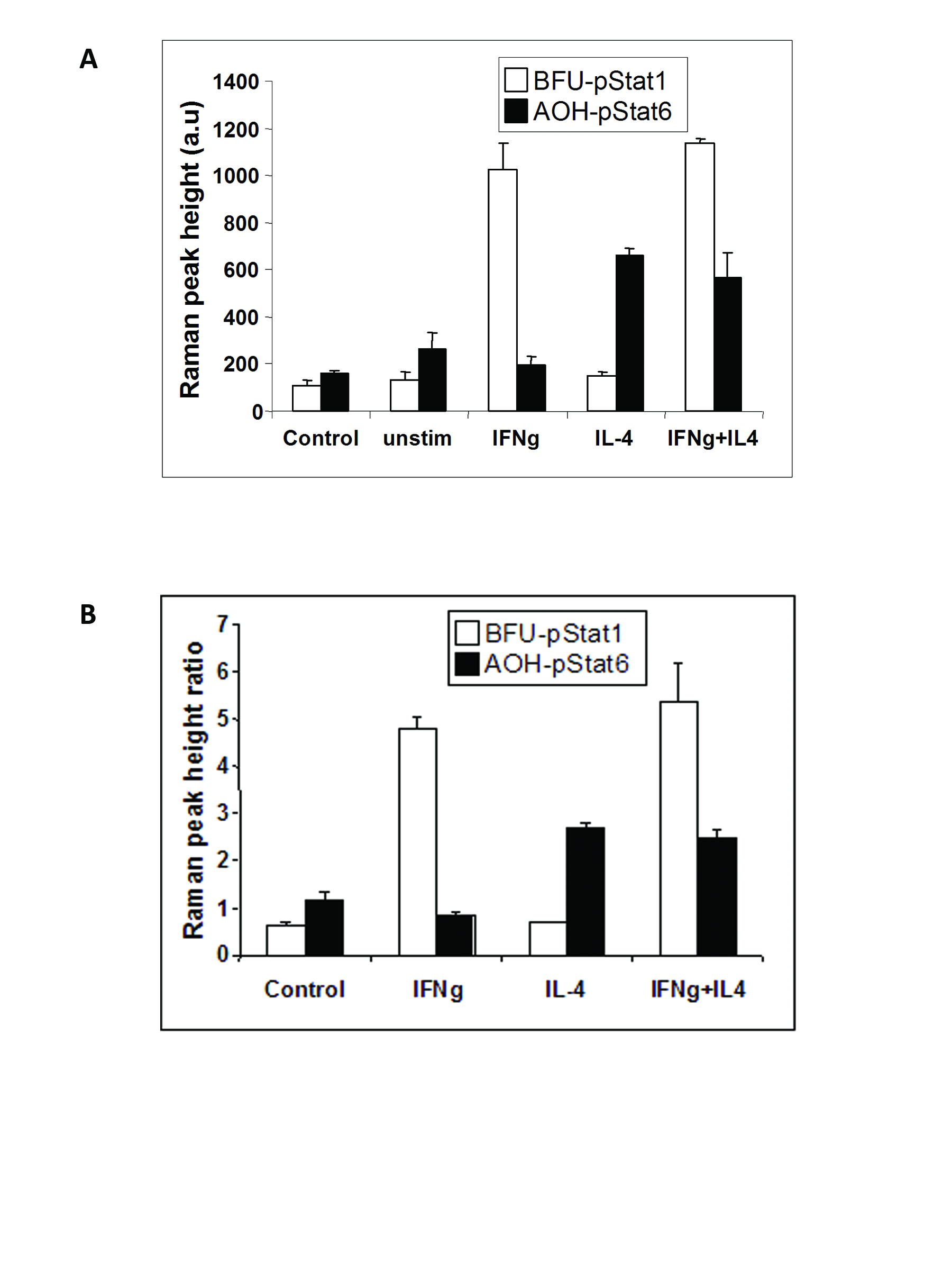

Supplement: Figure S5 — (1.28 MB TIF) [file pone.0005206.s005.tif]
